# Mapping and comparing French people’s positions regarding restrictive control policies: a pilot study

**DOI:** 10.1186/s13011-020-00267-5

**Published:** 2020-03-19

**Authors:** Sylvie Castanié, Maria Teresa Munoz Sastre, Lonzozou Kpanake, Etienne Mullet

**Affiliations:** 1 CERPPS, Maison de la recherche, Federal University of Toulouse, 5 allées Antonio Machado, 31058 Toulouse, cedex 9 France; 2 University of Québec – TELUQ, 5800, rue Saint-Denis, Bureau 1105, Montréal, Québec H2S 3L5 Canada; 3 Institute of Advanced Studies (EPHE), 17 bis, rue Quefes, Plaisance du Touch, 31830 Paris, France

**Keywords:** Control policies, Personal positions, Alcohol, Tobacco, Gaming, France

## Abstract

**Background:**

Public authorities resort to various control policies in order to curb the prevalence of unhealthy behaviors. As these policies can only succeed to the extent that people agree with them, this study mapped French people’s positions regarding restrictive control policies in general.

**Method:**

A sample of 344 adults (among them health professionals and lawyers) were presented with 54 vignettes depicting a control policy. Each vignette contained four pieces of information: the type of addictive behavior targeted (smoking, drinking, or gambling), the nature of preventive measures (e.g., information campaigns), the degree of regulative measures (e.g., prohibition to minors), and the severity of sanctions.

**Results:**

Through cluster analysis, eight qualitatively different positions were found: *Never acceptable* (9%), *Weak or moderate regulation* (5%), *Moderate regulation associated with strong prevention* (11%), *Strong or moderate regulation* (11%), *Strong regulation in association with strong prevention* (23%), *Moderate sanctions in association with strong prevention and moderate regulation* (9%), *Severe sanctions* (9%), and *Always acceptable* (9%). Some participants (14%) expressed no opinion at all.

**Conclusion:**

French people’s positions regarding control policies were extremely diverse. Regarding tobacco, however, one type of policy would likely be supported by a majority of people: Moderate regulation associated with at least a moderate level of prevention and low-level sanctions. Regarding alcohol, an acceptable position would be: Moderate regulation associated with at least a moderate level of prevention and high-level sanctions. Regarding gambling, an acceptable position would be: Strong regulation associated with at least a moderate level of prevention and low-level sanctions.

## Background

Smoking tobacco, drinking alcohol, and gambling are among the most popular recreational activities for Western Europeans and North Americans [1]. These recreational activities are certainly enjoyable in themselves, but they have heavy costs [1]. A regular smoker’s life expectancy is, for example, at least ten years shorter than that of a non-smoker [2]. Regular drinkers put not only their family at risk (by increasing the severity of domestic violence [3]), but also other community members [4]. Although a regular gambler’s health is not affected by gambling to the same extent as a smokers’ or drinkers’ health, gamblers face a considerably higher risk of suicide than non-gamblers [5].

Regarding tobacco, alcohol, gambling and other commodities that can lead to addiction, many governments are in an uncomfortable position because they are often the providers of these commodities and because, whether they are direct providers or not, they derive benefits from taxation on sales. Governments currently have no choice but to accept this discomfort because, if they were to prohibit tobacco or alcohol sales and gambling, the demand would result in full scale black markets, which would then enable consumers to satisfy their needs outside of the law. The same problem may arise if, instead of complete prohibition, governments increased the level of taxation of these commodities to such a point that many consumers no longer have access to them. The size of the black market in tobacco in a country is a direct function of tobacco taxes [6].

The demand for psychoactive substances is not a new phenomenon; substance use can be traced to prehistoric times [7]. Also, gambling is probably as old as the use of substances [8]. Both are universal. There is no chance that any prohibitive policy voted at one point in time by a group of representatives in a particular place can quickly put an end to a demand that is so deeply rooted in humanity. On the one hand, it is biology that has created highly sensitive receptors to these substances within our nervous system [7]. On the other hand, states, families and friends often endorse and promote gambling and substance consumption [9, 10].

In order to curb the frequency of behaviors that are associated with substance misuse and addiction, and are currently frowned upon and targeted by society, public authorities have issued various types of control policies [11]. Control policies can, however, only succeed in curbing the frequency of unhealthy behaviors to the extent that people understand the necessity of them and agree with their elements. When the public attitude towards a specific policy is negative, governments usually have no other choice than to withdraw their support for the policy [12, 13]. Negative views about control policies may also impair full implementation of and adherence to them [14]. In addition, to successfully counteract the efforts of unhealthy industries to sell more and more of their products, governments need strong support from civil society [1]. Many empirical studies have, therefore, been conducted on people’s attitudes toward control policies.

As for tobacco control policies, it has been found that non-smokers, ex-smokers, women, older participants, and participants with non-conservative political views were more supportive of them than smokers, men, younger participants, and participants with conservative views [15–18]. The impact of educational and socio-economic level on support has, however, been more difficult to assess [19]. Support for smoking control policies (notably, smoking bans in public areas) was usually higher sometime after their introduction than before their introduction, although support for measures seen as being intrusive, such as an increase in cost, was lower than support for measures seen as being more global, such as educational campaigns [15]. However, most people seem to consider that complete prohibition (namely, making smoking illegal) would not be feasible [20].

As for control policies relating to alcohol and gambling, the findings are very similar. Individuals who are fully aware of the health risks associated with alcohol consumption tend to support restrictive control policies, and more so if they also believe in their effectiveness [21]. A majority of people agree with the view that gambling advertisements should be banned during sporting events and that more information about the risks associated with gambling should be provided to children and adolescents [22]. Direct experience of harm from close relatives who exhibit risky behaviors was also positively associated with support for control policies [21, 23–25].

### The present study

The present pilot study examined and mapped, in a detailed way, people’s positions regarding restrictive control policies in general (not necessarily in the particular context of smoking, drinking, or gambling). It was conducted in France. In France, as in most other countries, people’s views on smoking, heavy drinking, and gambling are generally negative [26]. The Tobacco Control Scale (TCS) is a quantitative tool that allows precise assessment of a country’s efforts to curb the consumption of this substance [27]. France’s TCS score is 57 (out of 100), which places it midway between the United Kingdom (81) and Austria (33) [28]. As regards alcohol control policies and gambling control policies, France is in a similar position.

Most people in France, above all people who report a preference for left-wing parties, believe that the government is directly implicated each time a substance user’s health deteriorates. Perceived government responsibility is only slightly attenuated when systematic information campaigns exist or when the person’s level of consumption was immoderate. From the viewpoint of French people, it would only be in the hypothetical case of total prohibition of all substances that the government could be relieved from any responsibility [29].

At first glance, people’s positions can be ordered along a continuum ranging from (a) the consideration that risk-taking is a personal responsibility issue, in which case control policies are unwelcomed, to (b) the consideration that risk-taking behavior performed by individuals negatively affects the whole community, in which case control policies of all kinds are absolutely needed [30, 31]. As a result, several qualitatively distinct positions can be expected that reflect a person’s location on this continuum. For some participants, these positions would be principled ones; that is, they would be completely similar whether the context is alcohol intake, tobacco consumption, or gambling. For other participants, these positions would vary as a function of the behavior considered.

The first position to expect would be a position that expresses complete rejection of any kind of control policy [30]. The corresponding opposite position would be the view that the most acceptable control policies are those that establish the most constraining measures (e.g., aggressive prevention campaigns associated with strict regulation and severe sanctions). This position would be close to the “progressive prohibitionist” view regarding illicit drugs suggested by Goode [32].

Positions that would be popular among people indulging in one or more of the unhealthy behaviors would either be that (a) if controls are needed, the weakest ones would be the most acceptable, or (b) if strong regulation is needed, then sanctions for violations should not be too severe [18]. This position would be close to the “free-trade libertarian” view suggested by Goode [32]. A position that would be popular among people who do not believe in the efficiency of prevention and regulation would be that the only control policies acceptable are ones that establish severe sanctions for violations of regulative measures. In its disdain of prevention and regulation, this position is reminiscent of the “cultural conservative” view suggested by Goode [32]. This view would amount to believing that unhealthy behaviors are personal issues, but the community must nevertheless be protected from the few individuals that go too far and put others at risk.

Positions would also vary as a function of the type of recreational activity. For example, for the same group of people, very constraining control measures would be acceptable regarding drinking because drunk driving has very negative societal consequences, whereas the same constraining measures would not be acceptable regarding gambling because its negative consequences are perceived as lesser or inexistent [24].

In summary, this pilot study aimed to delineating people’s positions on policy control in general. As any policy is unlikely to succeed if people do not, at least to some extent, agree with it, it is important to identify, in a detailed way, the policies which individuals would like governments to implement and those that people would consider unacceptable because they are viewed as either too constraining or too relaxed. Several positions were expected that ranged from the most relaxed ones (e.g., weak regulation and weak sanctions) to the most constraining ones (e.g., high levels of prevention, strong levels of regulation and severe sanctions). For some participants, these would be principled positions and, for others, they would vary as a function of the commodity considered and whether or not they smoked, drank, and/or gambled in the past. In addition, the positions would be associated with participants’ characteristics (e.g., occupation or educational level) and with consumption habits.

## Method

### Participants

The participants were 344 adults (among them 5 experts in addiction, 13 physicians, 17 lawyers, and 91 other health professionals) who lived in southern France (the area of Pau) and were between 18 and 88 years old (*M* = 47.11, *SD* = 15.91). These participants were unpaid volunteers, recruited and tested by five research assistants trained in the technique used. The researchers contacted 400 people walking along city sidewalks of several big cities; of these, 218 (55%) participated. The main motive given for not participating was lack of time. The researchers also contacted physicians and nurses in medical centers, and lawyers and psychologists in their offices. Eleven per cent of participants were occasional smokers, 18% heavy smokers, 71% occasional drinkers, 13% heavy drinkers, 18% occasional gamblers, and 2% regular gamblers. Table 1 shows the demographic characteristics of the sample.

**Table 1:** Demographic Characteristics of the Sample. Distribution of Participants among the Clusters

	Cluster									
	Never	Weak or Moderate Reg.	Moderate Reg. & Prev.	Strong or Moderate Reg.	Strong Reg.	Moderate Sanct.	Strong Sanct.	Always	No Opinion	Total
Gender										
Male	25(8)	22(7)	44(13)	35(11)	75(23)	21(6)	19(6)	32(10)	54(16)	327
Female	66(9)	31(4)	66(9)	77(11)	164(23)	68(10)	77(11)	60(9)	96(14)	705
Age										
18–34 Years	19(7)	16(6)	31(11)	25(9)	58(22)	32(12)^a^	26(10)	15(6)^a^	45(17)	267
35–45 Years	26(11)	11(4)	25(10)	34(14)	72(29)^a^	18(7)	21(9)	8(3)^b^	31(13)	246
46–60 Years	22(8)	17(6)	26(10)	32(12)	68(26)^b^	28(11)^b^	19(7)	25(9)^bc^	30(11)	267
61+ Years	24(10)	9(4)	28(11)	21(8)	41(16)^ab^	11(4)^ab^	30(12)	44(17)^abc^	44(18)	252
Education										
Primary	31(8)	15(4)	39(10)	24(6)^ab^	71(19)^a^	23(6)	50(13)^a^	60(16)^ab^	68(18)	381
Secondary	29(9)	19(6)	40(12)	41(13)^a^	76(23)	36(11)	21(6)^a^	21(6)^a^	44(14)	327
Tertiary	31(10)	19(6)	31(10)	47(14)^b^	92(28)^a^	30(9)	25(8)	11(3)^b^	38(12)	324
Occupation										
Lay People	56(9)	36(5)	68(10)	63(10)	137(21)^ab^	51(8)^ab^	72(11)	74(11)	97(15)	654
Lawyers	4(8)	2(4)	4(8)	5(10)	19(37)^ac^	10(19)^a^	5(10)	0(0)	2(4)	51
Paramedical	22(10)	8(4)	25(12)	29(14)	56(26)^d^	13(6)^c^	10(5)	18(8)	32(15)	213
Psychologists	5(8)	7(12)	10(17)	8(13)	6(10)^cde^	12(20)^bc^	2(3)	0(0)	10(17)	60
Physicians	0(0)	0(0)	3(8)	7(18)	16(41)^be^	2(5)	5(13)	0(0)	6(15)	39
Addictionologists	4(27)	0(0)	0(0)	0(0)	5(33)	1(7)	2(13)	0(0)	3(20)	15
Political Orientation										
Extr. Left	29(21)^abc^	9(6)	14(10)	10(7)^a^	23(17)^a^	19(14)	9(7)	6(4)^a^	19(14)	138
Left	12(7)^a^	8(5)	22(13)	23(13)	34(19)	25(14)	10(6)	16(9)^b^	24(14)	174
Center	48(8)^b^	34(5)	68(11)	64(10)^b^	152(25)	41(7)	70(11)	49(8)^c^	95(15)	621
Right	0(0)^c^	2(3)	2(3)	14(24)^abc^	21(35)^a^	1(2)	5(8)	5(8)^d^	10(17)	60
Extr. Right	1(3)	0(0)	3(9)	0(0)^c^	9(27)	2(6)	2(6)	16(49)^a-d^	0(0)	33
Participant’s Level of the Addictive Behavior Mentioned in the Vignettes										
Absent	54(9)	22(4)^a^	34(6)^ab^	77(13)^a^	139(24)^a^	55(10)	43(8)	58(10)	91(16)	573
Moderate	24(7)	14(4)^b^	56(16)^a^	32(9)	85(25)^b^	22(6)	45(13)	26(8)	41(12)	345
Strong	13(11)	17(15)^ab^	20(18)^b^	3(3)^a^	15(13)^ab^	12(10)	8(7)	8(7)	18(16)	114
Type of Addiction Mentioned in the Vignettes										
Tobacco	40(12)	23(7)	43(12)^a^	43(12)^a^	71(21)	43(12)^a^	7(2)^a^	23(7)	51(15)	344
Alcohol	26(8)	19(6)	60(17)^b^	22(6)^ab^	74(21)	10(3)^ab^	65(19)^ab^	31(9)	37(11)^a^	344
Gambling	25(7)	11(3)	7(2)^ab^	47(14)^b^	94(27)	36(11)^b^	24(7)^b^	38(11)	62(18)^a^	344
Total	91 (9)	53 (5)	110 (11)	112 (11)	239 (23)	89 (9)	96 (9)	92 (9)	150 (14)	1032

Note: Figures with the same subscript are significantly different, *p* < .01. Figures in parentheses are percentages calculated for each row. The sample size was *N* = 344. The number of profiles of responses was *N* = 344 × 3 = 1032

As the aim of the study was to delineate how participants utilized the information provided in the vignettes, a community sample of participants with various types of personal and professional experience with regards to addiction, and with various levels of consumption of addictive commodities, was considered sufficient because the different possible positions were expected to be limited in number [33].

The study conformed to the ethical recommendations of the French Society of Psychology; that is, participants’ confidentiality was respected and informed consent was obtained. The study was approved by the Ethics Committee of the Federal University of Toulouse. Data gathering took place from July 2016 to May 2018.

### Material

The material was composed of three sets of 18 vignettes, each containing a short description of a control policy in terms of preventive measures, regulative measures and sanctions, and a response scale. The first set described smoking control policies. It was created by orthogonally combining the levels of the three factors that are shown in Table 2. For each factor, the levels were selected so that they encompassed at least one that was similar to current corresponding policies in France. An example of a vignette can be found in Appendix. The second and third sets described alcohol control policies and gambling control policies. For all sets, the response scale was an 11-point scale with two anchors labeled “Not at all acceptable” (0) and “Completely acceptable” (10).

**Table 2:** Levels of the Three Factors

Factor	Tobacco	Alcohol	Gambling
Levels of Prevention			
Lowest	No information campaigns		
Intermediate (Current policy)	Occasional information campaigns targeting populations at risk.		
Highest	Systematic information campaigns using the media. Information programs in schools. Personalized and professional phone service.		
Levels of Regulation			
Lowest	Smoking forbidden in public areas except bars and discotheques. Pricing: 5 euros. Sale prohibited to those less than 16 years of age.	Blood alcohol limit for drivers: 0.8 g/l. No control of selling points. Sale prohibited to those less than 16 years of age.	No control of player’s identity before access to gambling locations. No control over casinos and other similar services. Access limited to > 16 years of age.
Intermediate (Current policy)	Smoking forbidden in all public areas. Smoking areas allowed. Pricing: 7 euros. Sale prohibited to those less than 18 years of age.	Blood alcohol limit for drivers: 0.5 g/l. Control of selling points. Sale prohibited to those less than 18 years of age.	Systematic control of player’s identity before access to gambling locations. Control over installation of casinos and other similar services. Access limited to > 18 years of age.
Highest	Smoking forbidden in all public areas. Smoking areas not allowed. Pricing: 12 euros. Sale prohibited to those less than 21 years of age.	Blood alcohol limit for drivers: 0 g/l. Strict regulation of selling points. Restricted opening hours. Sale prohibited to those less than 21 years of age.	Control of player’s identity. Limitation of installation of casinos and similar services. Control of fraudulent activities. Prevention of pathological gambling. Access limited to > 21 years of age.
Level of Sanction			
Lowest (Current policy)	Fines in cases of violation	Fines in cases of violation	Temporary exclusion from gambling locations.
Highest	From fines to prison terms as a function of severity and recidivism.	From fines to mandatory care, to suspension of licenses, to prison terms as a function of severity and recidivism.	From mandatory care to prison terms as a function of severity and recidivism.

Three questions were used to assess the level of use of each substance, and the level of gambling: (a) How often do you smoke? (b) How often do you drink alcoholic beverages? and (c) How often do you gamble for money? A four-point scale was used for each question response: Never (0), From time to time (1), Often (2), and Daily (3).

### Procedure

The site of the study for lay people who were participating was a vacant room at the local university or the participant’s private home, and, for the professionals, a vacant room in the hospital or their office. Each person was tested individually. The procedure followed Anderson’s [33] recommendations for this type of study. The order of administration of the 54 vignettes was different from one participant to another and was randomly determined. Participants took 20 to 30 min to complete the ratings. No participant voiced any complaint about the number of vignettes or about the credibility of the proposed situations.

### Data analyses

As qualitatively different positions were expected, a cluster analysis, using the K-means procedure [34], was first applied to all sets of scenarios in order to detect different patterns of ratings. As each participant was given three series of 18 ratings on scenarios containing similar information that only differed regarding the context (tobacco, alcohol, or gambling), the total number of profiles of ratings analyzed was 3 × 344 = 1032. One requirement for comparing positions in each condition was to group participants using exactly the same clustering criteria.

A nine-cluster solution was retained because it was the one that produced the most interpretable findings. An overall analysis of variance (ANOVA) was conducted with a design of Cluster x Prevention x Regulation x Sanction (9 × 3 × 3 × 2). Cluster was a between-subjects factor and Prevention, Regulation, and Sanction were within-subjects factors. As the cluster effect and the three two-way interactions involving cluster were significant, nine separate ANOVAs were conducted at the cluster level, with a design of Prevention x Regulation x Sanction (3 × 3 × 2). The results are available from the corresponding author.

## Results

### Cluster analyses

The first cluster (9% of the profiles) was the expected *Never acceptable* cluster (not shown). All ratings were much lower than the middle of the acceptability scale. The overall mean rating was 1.60.

**Fig. 1:**
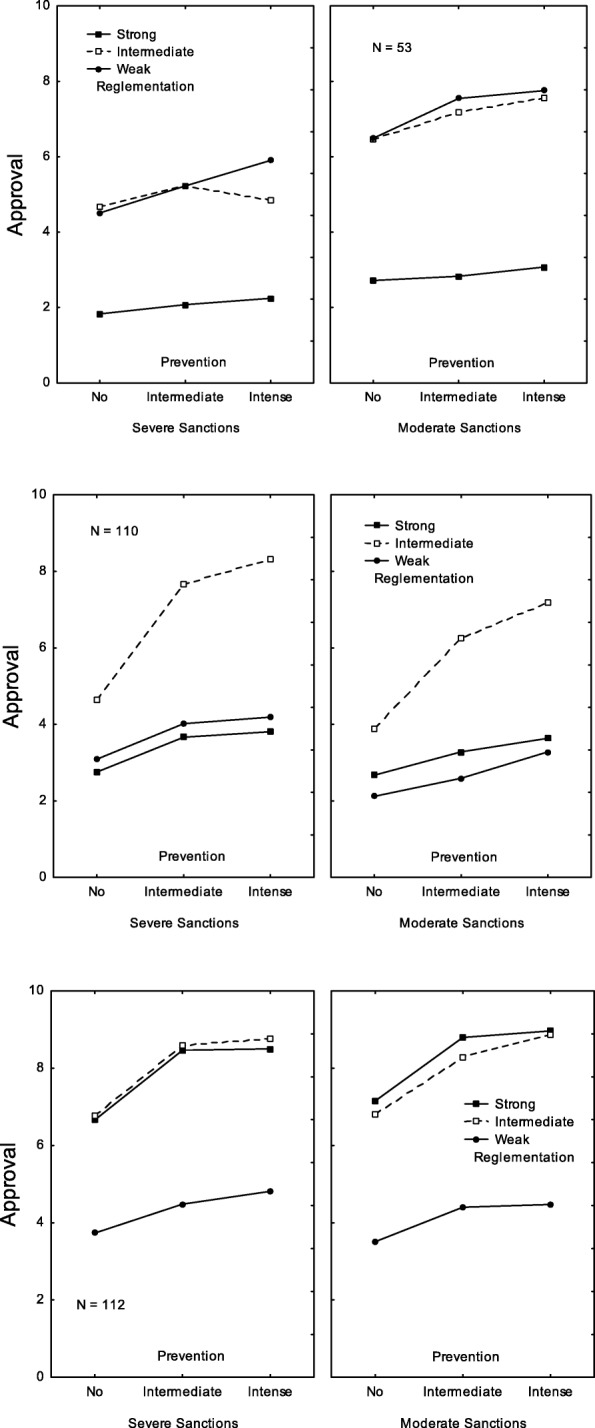
Clusters A. Pattern of ratings observed for three of the nine clusters: *Weak or moderate regulation* cluster (top panels), *Moderate regulation associated with strong prevention* cluster (center panels), and *Strong or moderate regulation* cluster (bottom panels). In each panel, the y-axis corresponds to the acceptability judgments, the x-axis bears the three levels of prevention, the three curves correspond to the three levels of regulation, and the two panels correspond to the two levels of sanctions

The second cluster (5%) was the expected cluster expressing preference for the weakest control policies. It was called *Weak or moderate regulation* because, as shown in Fig. 1 (top panels), ratings were much higher when regulation was weak (*M* = 5.24) or intermediate (*M* = 4.99) than when it was strong (*M* = 1.46). The effect of prevention was weaker when regulation was strong compared to both other cases.

The third cluster (11%) was called *Moderate regulation associated with strong prevention* because ratings were much higher (a) when regulation was intermediate (*M* = 5.32) rather than when it was weak (*M* = 2.22) or strong (*M* = 2.30), and (b) when prevention efforts were systematic (*M* = 4.07) rather than absent (*M* = 2.19) or occasional (*M* = 3.59).

The fourth cluster (11%) was called *Strong or moderate regulation* because ratings were much lower when regulation was weak (*M* = 3.23) compared to when it was strong (*M* = 6.39) or moderate (*M* = 6.17). In addition, ratings were higher when prevention efforts were intense (*M* = 6.39) or occasional (*M* = 6.17) rather than absent (*M* = 4.77). As shown in Fig. 1 (bottom panels), the effect of prevention was weaker when regulation was weak than in both other cases.

**Fig. 2:**
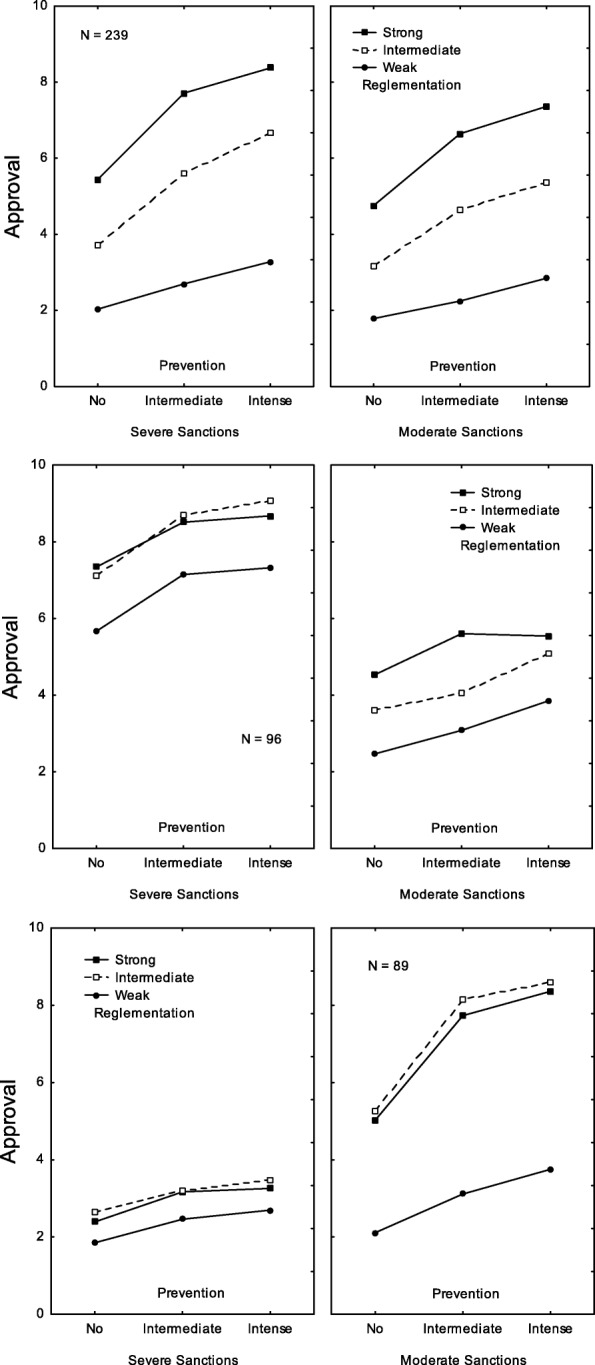
Clusters B. Pattern of ratings observed for three of the nine clusters: *Strong regulation in association with strong prevention* cluster (top panels), *Moderate sanctions in association with strong prevention and moderate regulation* cluster (center panels), and *Severe sanctions* cluster (bottom panels). In each panel, the y-axis corresponds to the acceptability judgments, the x-axis bears the three levels of prevention, the three curves correspond to the three levels of regulation, and the two panels correspond to the two levels of sanctions

The fifth cluster (23%) was the expected cluster expressing the highest acceptability level for policies that apply the most constraining measures. It was called *Strong regulation in association with strong prevention* because ratings were much higher (a) when regulation was strong (*M* = 5.71) compared to when it was weak (*M* = 1.48) or moderate (*M* = 3.86), and (b) when prevention efforts were systematic (*M* = 4.65) rather than absent (*M* = 2.49) or occasional (*M* = 3.93). As shown in Fig. 2 (top panels), the effect of prevention was weaker when regulation was weak (3.52–2.26 = 1.26) rather than when it was either moderate (6.33–4.03 = 2.30) or strong (7.44–5.20 = 2.24).

The sixth cluster (9%) was the expected cluster expressing preference for policies involving the weakest sanctions. It was called *Moderate sanctions in association with strong prevention and moderate regulation* because ratings were considerably lower when sanctions were potentially severe (*M* = 1.79) rather than not very severe (*M* = 4.79). Ratings were higher (a) when regulation was moderate (*M* = 4.21) or strong (*M* = 3.99) compared to when it was weak (*M* = 1.66), and (b) when prevention efforts were systematic (*M* = 4.02) rather than absent (*M* = 2.21) or occasional (*M* = 3.63). As shown in Fig. 2 (center panels), the effects of prevention and regulation and the Prevention x Regulation interaction were weaker when the level of sanction was potentially severe and much stronger in the alternative case.

The seventh cluster (9%) was the expected *Severe sanctions* cluster because ratings were considerably higher when sanctions were potentially severe (*M* = 6.73) rather than not very severe (*M* = 3.21). As shown in Fig. 2 (bottom panels), when sanctions were weak, the highest regulation level had the highest ratings, whereas, when sanctions were severe, the highest regulation level was rated the highest.

The eighth cluster (9%) was called *Always acceptable* (not shown). All ratings were much higher than the middle of the acceptability scale (*M* = 7.44). Finally, the ninth cluster (14%) was called *No strong opinion* (not shown). All ratings were close to the middle of the acceptability scale (*M* = 5.00 out of 10).

### Principled versus flexible positions

Principled stances are those that are relatively consistent across contexts, in contrast to flexible positions, which are more sensitive to contextual conditions. Table 3 shows the different sets of policies that were expressed by each participant in each of the three contexts. Twenty-two percent of participants systematically judged the strongest control policies as acceptable – *Severe sanction, Strong regulation, and Strong or moderate regulation* – in all three conditions, and 7% of participants systematically judged the weakest control policies as acceptable – *Moderate sanctions, Weak regulation, and Weak or moderate regulation* – in all three conditions. These participants expressed, therefore, principled positions. That is, they expressed positions that apply irrespective of the context, probably because they considered all the behaviors to be of similar risk.

**Table 3:** Type of Control Policy Preferred Across Commodities

Positions	*N*	%	Heavy Smokers	Heavy Drinkers	Regular Gamblers
Strong Control Policies in All Cases	77	22%	9%	4%	1%
Weak Control Policies in All Cases	25	7%	20%	16%	4%
Always Acceptable in All Cases	17	5%	18%	6%	12%
No Opinion in All Cases	15	4%	13%	20%	20%
Never Acceptable in All Cases	12	3%	0%	33%	0%
Subtotal	146	42%			
Weak Control Policies for Smoking and Strong Control Policies in Other Cases	20	6%	40%	15%	0%
Strong Control Policies for Gambling and Weak Control Policies in Other Cases	15	4%	47%	27%	0%
Weak Control Policies for Drinking and Strong Control Policies in Other Cases	13	4%	0%	8%	0%
No Opinion for Smoking and Strong Control Policies in Other Cases	13	4%	8%	8%	0%
No Opinion for Gambling and Weak Control Policies in Other Cases	12	3%	50%	17%	0%
Strong Control Policies for Drinking and Weak Control Policies in Other Cases	10	3%	20%	30%	0%
No Opinion for Gambling and Strong Control Policies in Other Cases	10	3%	0%	22%	0%
Subtotal	93	27%			
Other Configurations	105	31%			
Total	344	100%	18%	12%	2%

### Relationships between positions and the other variables

Some of the positions were significantly associated with participants’ characteristics. As shown in Table 1, the *Never acceptable* position was more often expressed by participants who reported preference for extreme right parties than by other participants. The *Weak or moderate regulation* and the *Moderate regulation and prevention* positions were more often expressed by participants who reported indulging in the behavior mentioned in the vignettes than by other participants. The *Strong or Moderate regulation* position, was more often expressed by more educated participants, by participants who reported preference for rights parties, and by participants who did not report indulging in the behavior mentioned than by other participants.

The *Strong regulation* position was less often expressed by oldest participants, by participants with primary education, by lay people and psychologists, by participants who reported preference for extreme left parties, and by participants who reported indulging in the behavior mentioned than by other participants. The *Moderate sanctions* position was more often expressed by lawyers and psychologists than by other participants. The *Strong sanctions* position was more often expressed by participants with primary education than by other participants. The *Always acceptable* position was more often expressed by the oldest participants, the participants with primary education, and the participants who reported preference for extreme right parties than by other participants.

Regarding alcohol, the *Strong or moderate regulation* and *Moderate sanctions* positions were less often found and the *Strong sanctions* position more often found than they were in the cases of tobacco or gambling. Regarding gambling, the *Moderate regulation and prevention* position was less often found than it was in the cases of tobacco or alcohol.

## Discussion

As expected, several significantly different positions ranging along an interpretable, although complex, continuum were found. These positions related, as expected, with participants’ demographic characteristics, the type of addictive behavior, and consumption habits. These findings are consistent with those of previous studies that showed that public opinion varied considerably towards leisure activity policies and that these views were associated with sociodemographic characteristics [26, 35, 36].

In 9% of cases, participants systematically disapproved of control policies that established strong sanctions (e.g., incarceration) in cases of a violation of current regulation. In contrast, they agreed with policies that established strong (or moderate) regulation, with the condition that these policies consider undertaking strong prevention efforts. This position--*Moderate sanctions*--was comparatively more common among psychologists and lawyers, who probably feared the psychological consequences of incarceration, than among younger participants, who probably felt more concerned with police controls than older participants. This position was less common when the policy applied to drinking, which consequences (e.g., drunk driving) are usually perceived as potentially severe for other people.

In 5% of cases, participants agreed with policies that did not establish too strong regulation. This was probably because, in cases of strong regulation, opportunities for violation would be higher, with the resulting disagreement with severe sanctions. This position--*Weak or moderate regulation*--was comparatively more common among participants who strongly indulged in the addictive behavior considered in the vignette, probably because they feared that too much regulation would limit access to their favorite commodity. It is the closest to the “free-trade libertarian” position suggested by Goode [32].

In 11% of cases, participants agreed with policies that established moderate regulation with the condition, however, that strong prevention efforts should also be considered. This position--*Moderate regulation associated with strong prevention*--was comparatively more common among participants who indulged in the addictive behavior considered, probably because they feared limited access to their favorite commodity. This position was probably founded on the idea that the *status quo* was the best option.

In another 11% of cases, participants showed a preference for policies that established potentially severe sanctions. They also agreed with policies that established strong regulative measures, without being very concerned with prevention efforts. This position--*Strong or moderate regulation--*was comparatively more common among participants who reported secondary or tertiary education (probably because it included higher levels of regulation than the preceding ones), those who reported a preference for right-wing parties, when the policy applied to tobacco or gambling (where consequences are usually perceived as less severe for other people than the consequences of excessive alcohol intake), and among participants who strongly indulged in the addictive behavior considered in the material (probably because they also feared limited access to their favorite commodity).

In 23% of cases, participants showed a preference for policies that established potentially severe sanctions. They fully agreed with policies that established strong regulation measures with the condition, however, that strong prevention efforts were considered. This position--*Strong regulation in association with strong prevention*--was comparatively more common among young adults and participants reporting a university degree (probably because it implied educational efforts), among physicians and lawyers (probably because it implied strong regulation measures), those who reported a preference for right-wing parties, and among participants who never indulged in the addictive behavior considered in the vignette (probably because they had no reason to fear limited access to the corresponding commodity). This position was probably founded on strong beliefs in the effectiveness of prevention and regulation. It was also the closest to the “progressive prohibitionist” position suggested by Goode [32].

In 9% of cases, participants rejected all policies that did not establish potentially severe sanctions. This position--*Severe sanctions--*was comparatively more common among participants who reported primary school education (probably because they had more punitive views) than more educated participants, and when the policy applied to drinking (probably because drinking is perceived as more consequential for others compared to smoking or gambling). This position was probably founded on some level of disbelief in the effectiveness of prevention and regulation. It was also the closest to the “cultural conservative” position suggested by Goode [32].

In a substantial number of cases, participants expressed either disagreement or full agreement with all policies. In 9% of cases, participants did not consider that policing was useful. This position--*Never acceptable--*was comparatively more common among people who reported a preference for extreme-left parties, probably because it implied radical views. It was not particularly associated with substance consumption or heavy gambling. This position was reminiscent of the “radical constructivist” position suggested by Goode [32]. In another 9% of cases, participants found all types of control policies acceptable. These participants probably considered that even minimal regulation was better than no regulation at all (e.g., free sale of tobacco at cost price). This position--*Always acceptable*--was comparatively more common among older participants (17%) (probably because they have, in their younger years, already experienced weak control policies), participants who reported primary school education (which was consistent with older age), and those who reported a preference for extreme-right parties (probably because of their tendency to respect authority).

For 42% of study participants, the same type of policy was considered the most acceptable in all cases, regardless of the commodity considered. For the remaining participants, however, the preferred type of policy varied as a function of the commodity considered. For example, fifteen participants believed that gambling should be strongly controlled, but smoking and drinking should not be strongly controlled. Unsurprisingly, 47% of these participants were heavy smokers, 27% were heavy drinkers, and no one was a regular gambler. Preference for a policy and actual consumption of substance were associated, but only in relation to the regulation component of the policy.

### Limitations

The main limitation of this study was that the sample was a convenience sample of professionals and lay people living in one area of France who agreed to respond to a time-consuming survey. It is likely that individuals who participated in the study had more available time compared to those who refused, which, in turn, might have influenced their viewpoints on leisure activities. However, this pilot study was not epidemiological in character. As indicated above, its aim was to map, as precisely as possible, people’s views regarding control policies, and not to determine the exact percentages of individuals in the whole population that hold these views. Future studies should, using a shortened version of our material, analyze the views of fully representative samples of French adults and compare them with the views of people from other part of France and people from other countries, namely from countries with different drug and gambling control policies.

## Conclusion

French people’s positions regarding control policies are extremely diverse. For a substantial minority (more than 40%), these positions are principled ones, that is, they apply irrespective of the domain considered (e.g., for 22%, the more restrictive policy was always preferred). For other people, these positions are more flexible.

The legislator’s task in this domain is therefore especially arduous. Any change in direction is likely to be met with opposition from one or another group of people. However, despite the great diversity of personal positions found, and their variation as a function of the commodity considered and as a function of people’s characteristics, it is possible to delineate some common ground.

Regarding tobacco, one type of policy is likely to be supported by a majority of people: Moderate levels of regulation (no smoking in public areas, pricing at 7 euros, and no sale to minors) associated with at least a moderate level of prevention and a low-level of sanctions (only fines). Unsurprisingly, this type of policy is consistent with current legislation. This finding is also consistent with that of Schumann et al. [37], showing that current and former smokers in Germany were less willing to support sanctions against smokers (e.g. refusing medical care to smokers). Further, there is one type of policy that would face most people’s opposition: Low-levels of regulation (smoking allowed in bars and discotheques, low pricing, and sale allowed to 16 year-olds) associated with an absence of prevention measures. This shows that people, in their majority, are certainly unwilling to return to tobacco policies that were in effect in the past.

Regarding alcohol, one type of policy would be supported by a majority of people: Moderate levels of regulation (blood alcohol limit of 0.5 g/l, control of selling points, and no sale to minors) associated with at least a moderate level of prevention and a high-level of sanctions (possible imprisonment in cases of violation). This type of policy is stricter than the current legislation. In addition, one position would face most people’s opposition: Low-levels of regulation (blood alcohol limit of 0.8 g/l, no control of selling points, and sale allowed to 16 year-olds) associated with an absence of prevention measures and a low-level of sanctions. This finding echoes those of previous studies showing that the general public in the United Kingdom and Australia were both more favorable towards alcohol policies with greater enforcement of sanctions (e.g. more police patrolling the streets) compared to policies restricting the availability of alcohol (e.g. increasing prices) [35, 36]. This would therefore suggest, for example, that people are most concerned by the high-level of mortality caused by traffic accidents [35].

Finally, regarding gambling, one policy would be supported by a majority of people: Strong levels of regulation (control of players’ identity, age limitation at 21, limited number of casinos, control of fraudulent activities, prevention of pathological gambling) associated with at least a moderate level of prevention and a low-level of sanctions (only fines). This policy is also stricter than current legislation. In addition, one policy would face most people’s opposition: Low-levels of regulation associated with an absence of prevention measures. Similar reluctant attitudes towards freedoms for gambling suppliers and consumers were found among the general public in the United Kingdom, who considered gambling as a criminal issue and dangerous to families, communities, and society as a whole [26].

In summary, a majority of participants expressed views that were either in agreement with current control policies in France or, in the case of alcohol consumption and gambling, would be open to stricter policies. If these findings can be replicated on a larger, representative sample of the general public it is anticipated that the government’s attempts to establish stricter tobacco and gambling control policies would be supported by a majority of people in France.

## Data Availability

All data collected is available and can be accessed by contacting the corresponding author.

## Appendix

### Example of scenario

In a neighboring country, the government has launched systematic information campaigns through the media and school programs on the risks associated with smoking. Free personalized and professional phone services have also been established.

Smoking is forbidden in all public areas, although smoking areas have been allowed. The price of a pack of cigarettes has been tariffed at 7 euros and the sale of tobacco is prohibited to those under 18 years of age.

In cases of violation of these rules, sanctions can range from fines to prison terms as a function of severity and recidivism.

If this policy was applied in your country, to what extent would you find it acceptable?

“Not at all acceptable” o---o---o---o---o---o---o---o---o---o---o “Completely acceptable”

## Abbreviations

TCS: Tobacco Control Scale
ANOVA: Analysis of variance
M: Mean
